# Relationship between pregnant women's combined exposure to heavy metals and their offspring's congenital heart defects in Lanzhou, China

**DOI:** 10.3389/fped.2024.1291076

**Published:** 2025-01-10

**Authors:** Lulu Chen, Yaqin Zhao, Jianhao Sun, Xinjuan Jiao, Zhenzhen Wu, Jian Wang, Jie Qiu, Baohong Mao, Qing Liu

**Affiliations:** ^1^ Gansu University of Chinese Medicine, Lanzhou, Gansu Province, China; ^2^ Gansu Provincial Maternity and Child-care Hospital, Lanzhou, Gansu Province, China; ^3^ Clinical Medical College, Yangzhou University, Yangzhou, Jiangsu Province, China

**Keywords:** congenital heart defects, heavy metals, combined exposure, pregnancy, maternal blood

## Abstract

**Background:**

Previous research has demonstrated that exposure to individual heavy metals elevates the incidence rate of congenital heart defects (CHDs). However, there is a paucity of data concerning the relationship between combined exposure to multiple heavy metals and the occurrence of CHDs. This study seeks to investigate the association between combined heavy metal exposure in pregnant women and the incidence of CHDs in their offspring in Lanzhou, China.

**Methods:**

We conducted a comprehensive review of the birth cohort study undertaken at our hospital from 2010 to 2012, with the objective of investigating the association between combined heavy metal exposure in pregnant women and the incidence of CHDs in their offspring. This analysis was performed utilizing a multifactorial conditional logistic regression model.

**Result:**

A nested case-control study was conducted involving 97 case groups and 194 control groups. The median concentrations of nickel (Ni), barium (Ba), lead (Pb), and titanium (Ti) in the blood of pregnant women were measured at 25.58 μg/L, 84.38 μg/L, 69.67 μg/L, and 304.65 μg/L, respectively. The research identified a significant correlation between the concentrations of Ni, Pb, and Ti in the blood of pregnant women and the risk of CHDs (*P* < 0.05). The optimal cut-off for heavy metals in pregnant women's blood was determined using the ROC curve. Levels below this threshold indicated low exposure, while levels at or above it indicated high exposure. In comparison to low exposure levels, high exposure to nickel (≥189.29 μg/L) in pregnant women was associated with a 2.098-fold increase in the risk of CHDs in their offspring (OR = 3.098, 95% CI: 1.322–7.260). Similarly, high lead exposure (≥86.70 μg/L) resulted in a 1.192-fold increase in the risk of CHDs in offspring (OR = 2.192, 95% CI: 1.021–4.707). Furthermore, high exposure to titanium (≥404.22 μg/L) was linked to a 3.065-fold increase in the risk of CHDs in offspring (OR = 4.065, 95% CI: 1.887–8.758). When compared to low exposure levels, the combined exposure to four heavy metals in the blood of pregnant women is linked to a 4.946-fold increased risk of CHDs in their offspring (OR= 5.946, 95% CI: 2.872–12.309). A significant correlation was observed between Ti exposure levels and the combined exposure levels of four heavy metals in pregnant women, with respect to the risk of isolated CHDs and multiple CHDs (*P* < 0.05). Additionally, high Ni exposure levels in pregnant women are associated with an increased risk of multiple CHDs (OR 4.321, 95% CI: 1.646–11.348).

**Conclusion:**

The cumulative exposure levels of Ni, Ba, Pb, and Ti in the blood of pregnant women are correlated with an elevated risk of CHDs in their offspring.

## 1 Introduction

Congenital heart defects (CHDs) pertain to anomalies in the structure and function of the heart that arise during embryonic development (1), with a neonatal prevalence ranging from approximately 0.8%–1.2% (2). CHDs represent one of the most prevalent congenital conditions and are the foremost cause of mortality attributable to birth defects (3), thereby imposing a substantial global disease burden (4). The etiology of CHDs is multifaceted, involving both genetic and environmental factors, either independently or in combination (5). Despite advancements in genetic and genomic methodologies, the underlying causes of only approximately 30% of CHDs cases have been elucidated to date (6).Environmental factors, although more complex, significantly contribute to the development of CHDs. Maternal conditions such as obesity and diabetes, as well as behaviors like smoking and exposure to chemical teratogens, are notable non-genetic risk factors (7–10). Additionally, toxic trace elements, including heavy metals and other environmental pollutants, are closely associated with an increased risk of CHDs (10). Environmental factors are more controllable and identifiable than genetic ones, so CHDs can be prevented by recognizing and avoiding these risks.

With the advancement of modern society and the enhancement of industrial capabilities, the severity of environmental degradation and industrial pollution has escalated. Consequently, heavy metal contamination has emerged as a predominant environmental issue. Individuals are increasingly at risk of ingesting heavy metals through contaminated food, water, and air (11). Heavy metals are typically defined as metals with a density equal to or greater than 4.5 g/cm^3^ (12). These metals are resistant to natural degradation, possess prolonged biological half-lives, and can bioaccumulate within food chains (13, 14), thereby potentially exacerbating the risk of various diseases affecting the gastrointestinal, respiratory, cardiovascular, renal, hematopoietic, and nervous systems (12, 15). Additionally, heavy metals pose significant threats to women's reproductive health; prolonged exposure has been associated with conditions such as endometriosis, endometrial cancer, menstrual irregularities, spontaneous abortion, preterm birth, and stillbirth (16–19). In addition, heavy metal pollution can also increase the risk of CHDs (20). Previous studies have shown that heavy metals such as nickel (Ni) (21), barium (Ba) (22), lead (Pb) (23, 24), titanium (Ti) (25), cobalt (Co) (26), and aluminum (Al) (27) are risk factors for CHDs. However, existing research has predominantly focused on the relationship between exposure to individual heavy metals and the risk of CHDs. There is a paucity of studies examining the association between combined exposure to multiple heavy metals and the risk of CHDs.

In real-world scenarios, humans are frequently exposed to multiple heavy metals simultaneously, as certain metals can either enhance or inhibit the absorption of others, leading to potential synergistic or antagonistic interactions among metal combinations (11, 28). For instance, Beyer demonstrated that co-exposure to arsenic (As), Pb, and manganese (Mn) can adversely affect the neurodevelopment of infants and young children, with As potentially acting as a synergist to Mn toxicity (29). Furthermore, another study identified an antagonistic interaction between Pb and mercury (Hg) that negatively impacts verbal learning, memory, and attention in children aged 7–14 years, it was found that high Hg exposure masked the adverse effects of Pb, which may be related to the mechanism of parallel competition between the two metals (30).

Ni, Ba, Pb, and Ti are prevalent heavy metals encountered in daily life. Prior research has demonstrated that exposure to Ni, Ba, Pb, and Ti poses significant threats to public health. Nevertheless, there is a paucity of both domestic and international studies investigating the correlation between combined exposure to these metals in pregnant women and the incidence of CHDs in their offspring. Notably, China possesses substantial reserves of Ni, Ba, Pb, and Ti. Therefore, utilizing a birth cohort from Lanzhou, China, this study employed a prospective nested case-control design to examine the exposure levels of Ni, Ba, Pb, and Ti during pregnancy. The research aimed to quantify the concentrations of Ni, Ba, Pb, and Ti in the blood of pregnant women and investigate the association between combined exposure to these metals and the risk of CHDs in their offspring. The findings are intended to contribute to the theoretical foundation for the prevention and treatment of CHDs, thereby enhancing maternal and child health.

## 2 Materials and methods

### 2.1 Study population

A study of a birth cohort was conducted in 2010–2012 at the Gansu Provincial Maternity and Child Care Hospital in Lanzhou, China (31). A total of 10,542 individuals from this birth cohort completed baseline questionnaires and provided blood samples. Based on the diagnosis of CHDs in singleton births, 97 mother-newborn pairs were designated as the case group. For the control group, 194 mother-newborn pairs were selected based on criteria that included healthy, full-term singleton births with normal birth weight, maternal age within two years of the case group, and residence in the same neighborhood, maintaining a 1:2 case-control ratio. Ultimately, a total of 291 mother-newborn pairs were included as study participants. Detailed information regarding the recruitment procedure has been provided in our previous research (25). This study was conducted with the informed consent of the pregnant women and received approval from the Medical Ethics Committee of Gansu Provincial Maternal and Child Health Hospital.

### 2.2 Classification of congenital heart disease

CHDs are classified into isolated CHDs and multiple CHDs based on the complexity of the cardiac malformations (32). Isolated CHDs denote the presence of a single type of heart malformation in fetuses, whereas multiple CHDs indicate the occurrence of two or more distinct types of heart malformations.

### 2.3 Questionnaire interview

Upon obtaining informed consent from the pregnant participants, trained investigators conducted face-to-face interviews to complete the “China Birth Cohort Study—Maternal Health Survey Questionnaire.” This questionnaire was collaboratively designed by the Gansu Provincial Maternal and Child Health Hospital and the Yale School of Public Health. Finally, we incorporated the following variables into our analysis of the study population: maternal age, Chinese Han ethnicity, educational level of pregnant women, monthly household income (CNY), pregnancy history, reproductive history, pre-pregnancy health education, early pregnancy reactions, hypertensive disorders during pregnancy, gestational diabetes, amount of postpartum bleeding (ml), newborn gender, and delivery method.

### 2.4 Blood collection, storage, and analysis

Blood samples from the study participants were collected prior to delivery during their hospital stay. Specifically, pregnant participants had 3 ml of venous whole blood collected from the antecubital vein in a fasting state during the morning. The collection was performed using a 7 ml Ethylenediaminetetraacetic acid (EDTA)-anticoagulated vacuum blood collection tube. Post-collection, the samples were mixed by inversion and subsequently stored at −80 °C. All relevant sample information was systematically recorded in an electronic database for biological samples.

Frozen blood samples were thawed at ambient temperature, and 0.2 ml aliquots were sequentially transferred into digestion vessels. Subsequently, 1 ml of 68% HNO_3_ and 0.2 ml of 40% H_2_O_2_ were added to each vessel. The samples were then placed in an oven and maintained at 175 °C for 6–8 h. Following the cooling period, the digested extracts were transferred to 10 ml test tubes and diluted to a final volume of 10 ml with deionized water. The solutions were mixed thoroughly and set aside for subsequent instrumental analysis. Subsequently, the concentrations of Ni, Ba, Pb, and Ti in maternal blood were quantified using inductively coupled plasma mass spectrometry (ICP-MS, ICAP RQ, Thermo Fisher, USA). The operational parameters for the ICP-MS are as follows: the peristaltic pump speed is set at 40.0 rpm; the auxiliary gas utilized is argon with a purity exceeding 99.999% and a flow rate of 0.8 L/min; the atomizing gas is argon with a flow rate of 1.0 L/min; the cooling gas is argon with a flow rate of 14.0 L/min. The sampling cone aperture measures 1.1 mm, while the intercepting cone diameter is 0.75 mm. High purity helium gas is employed as the collision gas at a flow rate of 4.1 L/min. The scanning mode is configured to peak jump, with a power setting of 1,400 W. The collection time is 10 ms, the sampling depth is 12 mm, and the scanning is performed three times. Each calibration of the ICP-MS instrument must adhere to the following criteria: In STD mode, the intensity of ^115^In should exceed 2.2 × 10^5^, the intensity of ^7^Li should be greater than 5.5 × 10^4^, the intensity of ^59^Co should surpass 1.0 × 10^5^, and the intensity of ^238^U should be above 3.3 × 10^5^. In KED mode, the intensity of ^59^Co should exceed 3.0 × 10^4^, the Ce oxide ratio (155/140) should be less than 0.02, and the Co/ClO ratio should be greater than 18. Internal standards, specifically ^103^Rh, ^45^Sc, ^72^Ge, ^115^In, ^150^Re and ^7^Li were employed for quality control purposes. By constructing the standard calibration curves for Ni, Ba, Pb, and Ti, the obtained correlation coefficients *R*^2^ were 0.9999, 0.9999, 1.0000, and 0.9998, respectively. These values indicate that the correlation coefficients *R*^2^ for the standard calibration curves of Ni, Ba, Pb, and Ti all exceed 0.9990. These results demonstrate a strong linear relationship within the specified range, confirming the suitability of the sample for measurement. The standard deviation of the counts per second (CPS) values for Ni, Ba, Pb, and Ti, as well as the ratio of CPS values to the internal standard CPS values, was calculated. The detection limit was determined by taking the ratio of three times the standard deviation to the slope of the linear equation. The detection limits for Ni, Ba, Pb, and Ti were 0.052 μg/L, 0.206 μg/L, 0.008 μg/L, and 0.071 μg/L, respectively. The quantitation limits for Ni, Ba, Pb, and Ti were 0.173 μg/L, 0.686 μg/L, 0.025 μg/L, and 0.240 μg/L, respectively, using the ratio of ten times the standard deviation to the slope of the linear equation. A value of zero is recorded when the concentration falls below the detection threshold. The detection rates of Ni, Ba, Pb, and Ti in the blood of pregnant women were 67.69%, 99.67%, 100.00%, and 100.00%, respectively.

### 2.5 Statistical analysis

The data were processed and analyzed utilizing SPSS version 25.0 statistical software (IBM, Chicago, USA). Categorical variables are presented as percentages (%). Quantitative data adhering to a normal distribution are reported as the mean ± standard deviation, whereas quantitative data not conforming to a normal distribution are represented by interquartile ranges. Univariate analysis between the case and control groups was conducted using chi-square tests for categorical variables. For continuous variables that adhered to a normal distribution, *t*-tests were utilized, whereas Mann–Whitney *U* test were applied to those that did not conform to a normal distribution.

Given the absence of established reference ranges for maternal blood levels of Ni, Ba, Pb, and Ti, this study employed receiver operating characteristic (ROC) curve analysis, guided by methodologies from prior research (25, 33), to determine the optimal threshold values for the concentrations of these elements in maternal blood. The optimal critical values for the concentrations of Ni, Ba, Pb, and Ti, in maternal blood were determined to be 189.29 μg/L, 213.84 μg/L, 86.70 μg/L, and 404.22 μg/L, respectively. Based on these critical values, the concentrations of Ni, Ba, Pb, and Ti in the blood of pregnant women were categorized into low exposure and high exposure groups. The level of heavy metal exposure was then quantified using a scoring system, where high exposure was assigned a score of 2 points and low exposure was assigned a score of 1 point. Calculate the cumulative score for four heavy metals (ranging from 4 to 8 points). By plotting the operating characteristics of the subjects, the optimal threshold for the cumulative exposure score of Ni, Ba, Pb, and Ti in pregnant women was determined. Subsequently, subjects were categorized based on this optimal threshold. The impact of combined exposure on the incidence of CHDs was then analyzed. Due to the selection of the control group being based on the matching of maternal age and place of residence in the case group, a conditional logistic regression model was employed to examine the relationship between the combined exposure levels of the four heavy metals and the risk of various types of CHDs.

## 3 Results

### 3.1 Characteristics of participants

A total of 291 mother-infant pairs meeting the inclusion criteria were enrolled in this study. Among these, 97 cases involved CHDs, comprising 43 cases of isolated CHDs and 54 cases of multiple CHDs. The control group consisted of 194 mother-infant pairs, maintaining a 1:2 ratio with the case group. Table 1 presents the distribution of general characteristics of postpartum women in both the case and control groups. Statistically significant differences were observed between the groups in terms of hypertensive disorders during pregnancy, gestational diabetes, postpartum hemorrhage, and delivery method (*P* < 0.05).

**Table 1:** Descriptive characteristics of the study sample*.

Variable	Cases *N* = 97(%)	Control *N* = 194 (%)	Chi square	*P*-value
Maternal age (years, *n*)			3.034	0.082
*n* ≤ 35	80 (82.5)	174 (89.7)		
*n* > 35	17 (17.5)	20 (10.3)		
Chinese Han population			2.445	0.118
Yes	90 (92.8)	189 (97.4)		
No	7 (7.2)	5 (2.6)		
Educational level of pregnant women			0.122	0.727
Below university	65 (67.0)	126 (65.0)		
University or above	32 (33.0)	68 (35.0)		
Monthly household income (CNY, *n*)			0.342	0.559
*n* ≤ 3,000	57 (58.8)	107 (55.2)		
*n* > 3,000	40 (41.2)	87 (44.8)		
Pregnancy history			0.030	0.863
Yes	36 (37.1)	70 (36.1)		
No	61 (62.9)	124 (63.9)		
Reproductive history			0.070	0.791
Yes	31 (32.0)	65 (33.5)		
No	66 (68.0)	129 (66.5)		
Pre-pregnancy health education			0.342	0.559
Yes	45 (46.4)	83 (42.8)		
No	52 (53.6)	111 (57.2)		
Early pregnancy reaction			0.249	0.618
Yes	50 (51.5)	106 (54.6)		
No	47 (48.5)	88 (45.4)		
Hypertensive disorder during pregnancy			15.608	<0.001
Yes	9 (9.3)	0 (0.0)		
No	88 (90.7)	194 (100.0)		
Gestational diabetes			12.253	0.001
Yes	6 (6.2)	0 (0.0)		
No	91 (93.8)	194 (100.0)		
Amount of Postpartum bleeding (ml, *n*)			7.908	0.005
*n* < 500	87 (89.7)	189 (97.4)		
*n* ≥ 500	10 (10.3)	5 (2.6)		
Newborn Gender			0.445	0.505
male	46 (47.4)	84 (43.3)		
female	51 (52.6)	110 (56.1)		
Delivery method			14.932	<0.001
Vaginal delivery	41 (42.3)	128 (66.0)		
Caesarean section	56 (57.7)	66(34.0)		

*Chi-square tests.

### 3.2 Comparison of Ni, Ba, Pb, and Ti concentrations in pregnant women's blood between groups

As illustrated in Table 2, the median concentrations of Ni, Ba, Pb, and Ti in the blood of pregnant women were measured at 25.58 μg/L, 84.38 μg/L, 69.67 μg/L, and 304.65 μg/L, respectively. As illustrated in Table 3, the concentrations of Ni and Pb in the blood of pregnant women did not exhibit significant differences between the case group and the control group (*P* > 0.05). However, the concentrations of Ba and Ti in the blood of pregnant women were significantly different between the case group and the control group (*P* < 0.05).

**Table 2:** The concentrations of Ni, Ba, Pb and Ti in the blood of pregnant women.

Heavy metal	Mean ± Standard deviation (μg/L)	P50 (μg/L)	P25–P75 (μg/L)
Ni	110.80 ± 295.50	25.58	0.00, 108.24
Ba	212.68 ± 791.04	84.38	55.45, 158.58
Pb	97.22 ± 282.03	69.67	44.64, 97.35
Ti	441.57 ± 822.57	304.65	207.13, 433.43

**Table 3:** Comparison of Ni, Ba, Pb and Ti concentrations in pregnant women's blood between groups*.

Heavy metal	Case (μg/L)		Control (μg/L)		*Z*	*P*-value
	P_50_	P_25_–P_75_	P_50_	P_25_–P_75_		
Ni	38.96	0.00, 192.57	25.58	0.00, 92.85	−1.605	0.108
Ba	108.99	51.69, 256.12	84.38	55.85, 135.79	−2.415	0.016
Pb	79.65	44.76, 113.84	69.67	43.53, 86.83	−1.706	0.088
Ti	371.91	188.85, 659.15	302.41	217.34, 389.95	−2.485	0.013

*Mann–Whitney *U* test.

### 3.3 Effect of single/combined exposure levels of Ni, Ba, Pb, and Ti in pregnant women on the risk of CHDs in their offspring

Our study identified a significant correlation between the concentrations of Ni, Pb, and Ti in the blood of pregnant women and the risk of CHDs in their offspring. Specifically, as illustrated in Table 4, compared to low exposure levels, elevated concentrations of Ni in maternal blood were associated with a 2.098-fold increase in the risk of CHDs in offspring (aOR^1^: 3.098, 95% CI: 1.322–7.260; *P* < 0.05). Furthermore, high exposure levels of Pb in maternal blood were linked to a 1.192-fold increase in the risk of CHDs in offspring (aOR^1^: 2.192, 95% CI: 1.021–4.707; *P* < 0.05). Elevated concentrations of Ti in maternal blood are associated with a 3.065-fold increased risk of CHDs in offspring (aOR^1^: 4.065, 95% CI: 1.887–8.758; *P* < 0.05). Similarly, high levels of Ba in the blood of pregnant women are linked to an increased risk of CHDs compared to low exposure levels (cOR: 4.268, 95% CI: 1.938–9.403). However, this association was not statistically significant after further adjustments (*P* > 0.05).

**Table 4:** Effect of single/combined exposure levels of Ni, Ba, Pb and Ti in pregnant women on the risk of CHDs in their offspring.

Heavy metals		Low	High	*P*-value
Ni	Concentration range (μg/L)	<189.29	≥189.29	
	Cases (*n*)/controls (*n*)	68/172	29/22	
	c*OR* (95% *CI*)	Reference	4.055 (1.912–8.599)	<0.001
	a*OR*^1^ (95% *CI*)	Reference	3.098 (1.322–7.260)	0.009
Ba	concentration range (μg/L)	<213.84	≥213.84	
	Cases (*n*)/controls (*n*)	68/175	29/19	
	c*OR* (95% *CI*)	Reference	4.268 (1.938–9.403)	<0.001
	a*OR*^1^ (95% *CI*)	Reference	1.422 (0.537–3.766)	0.479
Pb	concentration range (μg/L)	<86.70	≥86.70	
	Cases (*n*)/controls (*n*)	52/146	45/48	
	c*OR* (95% *CI*)	Reference	2.353 (1.207–4.587)	0.012
	a*OR*^1^ (95% *CI*)	Reference	2.192 (1.021–4.707)	0.044
Ti	concentration range (μg/L)	<404.22	≥404.22	
	Cases (*n*)/controls (*n*)	51/151	46/43	
	c*OR* (95% *CI*)	Reference	4.317 (2.191–8.504)	<0.001
	a*OR*^1^ (95% *CI*)	Reference	4.065 (1.887–8.758)	<0.001
Combined exposure	Range (points)	<5.5	≥5.5	
	Cases (*n*)/controls (*n*)	56/168	41/26	
	c*OR* (95% *CI*)	Reference	4.767 (2.599–8.745)	<0.001
	a*OR*^2^ (95% *CI*)	Reference	5.946 (2.872–12.309)	<0.001

*aOR*^1^, adjusted hypertensive disorder in pregnancy, Gestational diabetes, Postpartum bleeding, delivery method and the other three heavy metals besides the studied heavy metals.

*aOR*^2^, adjusted hypertensive disorder in pregnancy, Gestational diabetes, Postpartum bleeding and delivery method.

The comprehensive exposure scores for Ni, Ba, Pb, and Ti in the blood of pregnant women predominantly fall within the range of 4–5 points, representing 76.98% of the sample. The mean score for the case group is 5.54 ± 1.392, which is significantly higher than that of the control group, which is 4.68 ± 0.776. By analyzing the working characteristics of the subjects, the optimal cutoff value for determining the combined exposure level scores of Ni, Ba, Pb, and Ti in maternal blood is identified as 5.5. A combined exposure level score of less than 5.5 is classified as low exposure, whereas a score of 5.5 or greater is classified as high exposure. A multivariate conditional logistic regression model was developed to examine the association between combined exposure levels of Ni, Ba, Pb, and Ti in maternal blood and the risk of CHDs. The findings indicate that elevated combined exposure levels of these four heavy metals in pregnant women significantly increase the risk of CHDs in their offspring. Specifically, high exposure levels of these metals in maternal blood were associated with a 4.946-fold increase in the risk of CHDs compared to low exposure levels (aOR^2^: 5.946, 95% CI: 2.872–12.309; *P* < 0.05). Detailed information can be found in Table 4.

### 3.4 Multivariate logistic regression model of single/combined heavy metal exposure of pregnant women on the risk of different types of CHDs in their offspring

Utilizing a conditional logistic regression model, this study investigates the association between the concentrations of four heavy metals in maternal blood and the risk of various CHDs. The findings indicate a significant correlation between Ti exposure levels, as well as the combined exposure levels of the four heavy metals in pregnant women, and the risk of different types of CHDs (*P* < 0.05). Elevated exposure levels of Ni in pregnant women are associated with an increased risk of multiple CHDs (aOR: 4.321, 95% CI: 1.646–11.348). However, no statistically significant correlation was observed between Ni exposure and the risk of isolated CHDs (*P* > 0.05). Furthermore, this study found no significant association between exposure levels of Ba and Pb in pregnant women and the risk of various types of CHDs (*P* > 0.05). For detailed information, please refer to Table 5.

**Table 5:** A multivariate logistic regression model of single/combined heavy metal exposure of pregnant women on the risk of different types of CHDs in their offspring.

Heavy metal		Controls (*n*)	Isolated		Multiple	
			Cases (*n*)	a*OR*	Cases (*n*)	a*OR*
Ni^a^	Low	172	34	Reference	34	Reference
	High	22	9	2.048 (0.536–7.818)	20	4.321 (1.646–11.348)
	*P*-value			0.294		0.003
Ba^a^	Low	175	29	Reference	39	Reference
	High	19	14	3.010 (0.815–11.136)	15	0.906 (0.265–3.101)
	*P*-value			0.098		0.875
Pb^a^	Low	146	22	Reference	30	Reference
	High	48	21	2.651 (0.852–8.245)	24	2.229 (0.876–5.674)
	*P*-value			0.092		0.093
Ti^a^	Low	151	22	Reference	29	Reference
	High	43	21	4.464 (1.461–13.636)	25	4.507 (1.756–11.571)
	*P*-value			0.009		0.002
Combined exposure^b^	Low	168	26	Reference	30	Reference
	High	26	17	7.061 (2.473–20.160)	24	5.493 (2.332–12.939)
	*P*-value			<0.001		<0.001

^a^ Adjusted hypertensive disorder in pregnancy, Gestational diabetes, Postpartum bleeding, delivery method and the other three heavy metals besides the studied heavy metals.

^b^ Adjusted hypertensive disorder in pregnancy, Gestational diabetes, Postpartum bleeding and delivery method.

## 4 Discussion

The detrimental impact of heavy metals on the human body is multifaceted, encompassing the adverse effects of individual metals on multiple organs and systems, as well as the more intricate consequences arising from the synergistic interactions of multiple metals. Combined exposure to multiple heavy metals poses a greater risk to human health compared to exposure to a single metal. Notably, organisms in the environment are typically subjected to a mixture of metal contaminants, and the cumulative effects of these exposures can result in adverse health outcomes even when the concentrations of individual metals are below their respective no-effect thresholds (34). Utilizing a single metal exposure model, this study identified a positive correlation between the blood levels of Ni, Pb, and Ti in pregnant women and the incidence of CHDs in their offspring. The risk of CHDs was observed to escalate proportionally with increasing exposure levels of Ni, Pb, and Ti. Conversely, no significant association was found between Ba exposure levels and the risk of CHDs in the offspring. The integrated exposure model revealed a correlation between the combined blood levels of Ni, Ba, Pb, and Ti in pregnant women and an elevated risk of CHDs and their various subtypes in their offspring.

### 4.1 Ni, Ba, Pb, Ti and CHDs

Ni is capable of traversing the placental barrier, thereby entering fetal circulation and amniotic fluid (35). Prior animal studies have demonstrated that excessive exposure to Ni exerts teratogenic effects on cardiac development (36). A case-control study conducted by Zhang indicated that elevated levels of Ni in maternal blood are associated with an increased risk of CHDs and are correlated with various types of CHDs (21). In this study, we observed that, relative to low exposure levels, elevated concentrations of Ni in the blood of pregnant women are associated with a 2.098-fold increase in the risk of CHDs in their offspring. Furthermore, high exposure levels of Ni during pregnancy were found to elevate the risk of multiple CHDs, corroborating findings from previous research.

Concerning the effects of Ba on reproduction and development, animal studies have demonstrated that elevated levels of Ba exposure may result in spontaneous abortion, fetal growth restriction, and fetal death (37, 38). However, there is no consensus regarding the association between Ba exposure and CHDs. A multicenter case-control study conducted by Zhang in China identified a dose-dependent relationship between maternal Ba exposure and the risk of CHDs in offspring (22). A case-control study conducted by Zierler in the United States found no statistically significant association between the barium content of drinking water consumed by pregnant women and an increased incidence of CHDs in their offspring (39). This study identified a statistically significant difference in blood Ba levels between pregnant women in the case group and those in the control group. However, upon further adjustment for confounding variables, no significant correlation was observed between blood Ba exposure levels in pregnant women and the risk of CHDs. No significant association was observed between Ba levels in the blood of pregnant women and the risk of various CHDs. The inconsistencies in these findings may be attributed to variations in sample size, study design, and exposure measurement methodologies. Consequently, further research is warranted to elucidate the potential impact of Ba on CHDs risk and to understand the underlying mechanisms of action.

Pb is capable of crossing the placental barrier, resulting in fetal blood lead levels that are positively correlated with maternal blood lead levels. Previous studies have indicated that maternal exposure to lead may be associated with adverse pregnancy outcomes. Results from a case-control study conducted in Iran indicated that mothers of infants with CHDs exhibited higher blood Pb concentrations compared to mothers of healthy infants (40). Similarly, a case-control study in China identified a significant association between elevated Pb concentrations in the hair of pregnant women and an increased risk of CHDs in their offspring (41). The findings of the present study demonstrate that elevated Pb exposure levels in the blood of pregnant women can increase the risk of CHDs in their offspring by a factor of 1.192, corroborating previous research.

Previous research has indicated a correlation between Ti exposure and negative pregnancy outcomes. Jin conducted a nested case-control study in regions with significant Ti exposure and discovered that elevated total blood Ti levels in pregnant women were linked to an increased risk of delivering low birth weight infants (42). Nevertheless, there is currently a paucity of studies investigating the relationship between Ti exposure and CHDs both domestically and internationally. This study demonstrated that elevated levels of Ti in the blood of pregnant women are associated with a 3.065-fold increase in the risk of CHDs in their offspring, and that the concentration of Ti is correlated with various types of CHDs.

### 4.2 Combined exposure of Ni, Ba, Pb, and Ti with CHDs

Currently, research on the health effects of heavy metals predominantly concentrates on the impact of individual heavy metal exposure. However, in real-world environments, exposure to multiple heavy metals simultaneously is common, and the health effects of two or more heavy metals may interact synergistically or antagonistically. For instance, a study involving individuals demonstrated that simultaneous exposure to elevated levels of Pb and cadmium (Cd), or to elevated levels of Pb coupled with reduced levels of selenium (Se), was associated with an increased risk of CHDs. These findings suggest the potential for a synergistic effect in the pathogenesis of CHDs attributable to blood Pb and blood Cd, while indicating the possibility of an antagonistic effect in the pathogenesis of CHDs related to blood Pb and blood Se (23). Wang conducted a study examining the combined exposure to As, Cd, Hg, Pb, and Mn and its association with the risk of CHDs in pregnant women. The findings suggest that the risk of CHDs increases with the level of the combined exposure to these elements (6).

The findings of this study indicate that the cumulative exposure to Ni, Ba, Pb, and Ti in the blood of pregnant women is associated with an elevated risk of CHDs. Specifically, when compared to low exposure levels, the high combined exposure to these four heavy metals in the maternal blood was found to increase the risk of CHDs in the offspring by a factor of 4.946. Compared to the individual exposure to Ni, Ba, Pb, and Ti regarding the risk of CHDs, combined exposure significantly elevated the risk, suggesting a possible synergistic effect among these four heavy metals. Furthermore, this study identified an association between combined exposure levels of these heavy metals in the blood of pregnant women and the risk of developing various types of CHDs.

### 4.3 Strengths and limitations

Firstly, maternal blood samples were chosen over urine and hair due to their higher representativeness. Secondly, this study not only examined the impact of individual exposures to Ni, Ba, Pb, and Ti on the risk of CHDs but also investigated the relationship between combined exposures to these metals and the risk of CHDs. Furthermore, CHDs were categorized into isolated and multiple types, and the effects of heavy metal exposure on the risk of these distinct CHDs types were analyzed. However, this study employed a scoring method based on Roberts' research methodology to analyze the combined exposure effect (33), this approach may not sufficiently account for potential interactions between chemicals. Although Bayesian Kernel Machine Regression (BKMR) and Weighted Quantile Sum (WQS) regression were not employed in the present study, we posit that these methodologies hold significant value for a more comprehensive understanding of the complex health effects associated with environmental chemicals. We intend to explore the application of BKMR and WQS in future research endeavors and anticipate that this will yield further insights. It is important to note that this research employed a case-control study design, which does not permit elucidation of the mechanisms through which heavy metals influence CHDs risk. Consequently, additional population studies and experimental investigations are required to elucidate the relationship between combined exposure to heavy metals and CHDs, as well as to uncover the potential pathogenic mechanisms involved.

## 5 Conclusion

Overall, the concentrations of Ni, Pb, and Ti in the blood of pregnant women exhibit a positive correlation with the incidence of CHDs in their offspring, with the risk progressively increasing in tandem with higher exposure levels of these metals. Additionally, the combined exposure to Ni, Ba, Pb, and Ti in maternal blood is associated with an elevated risk of CHDs and is linked to various types of these defects. Further research with larger sample sizes is required to substantiate these findings and to investigate the underlying mechanisms by which heavy metal exposure may induce CHDs.

## Data Availability

The raw data supporting the conclusions of this article will be made available by the authors, without undue reservation.
